# Dynamic and Static Mechanical Properties of Crosslinked Polymer Matrices: Multiscale Simulations and Experiments

**DOI:** 10.3390/polym10070792

**Published:** 2018-07-19

**Authors:** Daria V. Guseva, Vladimir Yu. Rudyak, Pavel V. Komarov, Boris A. Bulgakov, Alexander V. Babkin, Alexander V. Chertovich

**Affiliations:** 1 Faculty of Physics, Lomonosov Moscow State University, Leninskie gory, 1-2, 119991 Moscow, Russia; guseva@polly.phys.msu.ru (D.V.G.); vurdizm@gmail.com (V.Y.R.); 2 Department of General Physics, Tver State University, Sadovyj per., 35, 170002 Tver, Russia; pvkomarov@gmail.com; 3 Nesmeyanov Institute of Organoelement Compounds, Russian Academy of Sciences, Vavilova st., 28, 119991 Moscow, Russia; 4 Institute of New Carbon Materials and Technologies, Leninskie gory, 1-11, 119991 Moscow, Russia; bbulgakov@gmail.com (B.A.B.); ababkin@inumit.ru (A.V.B.); 5 Faculty of Chemistry, Lomonosov Moscow State University, Leninskie gory, 1-3, 119991 Moscow, Russia

**Keywords:** polymer matrix, phthalonitrile resin, dynamic mechanical analysis, conversion, sol-gel transition

## Abstract

We studied the static and dynamic mechanical properties of crosslinked polymer matrices using multiscale simulations and experiments. We continued to develop the multiscale methodology for generating atomistic polymer networks, and applied it to the case of phthalonitrile resin. The mechanical properties of the resulting networks were analyzed using atomistic molecular dynamics (MD) and dissipative particle dynamics (DPD). The Young’s and storage moduli increased with conversion, due both to the appearance of a network of covalent bonds, and to freezing of degrees of freedom and lowering of the glass transition temperature during crosslinking. The simulations’ data showed good quantitative agreement with experimental dynamic mechanical analysis measurements at temperatures below the glass transition. The data obtained in MD and DPD simulations at elevated temperatures were conformable. This makes it possible to use the suggested approach for the prediction of mechanical properties of a broad range of polymer matrices, including ones with high structural heterogeneity.

## 1. Introduction

Composites, namely, carbon fiber reinforced plastics (CFRP), substitute traditional metal materials in many applications, for example, in the production of parts for aircrafts, bridges, and automobiles. However, despite a variety of composite advantages that have made possible a wide range of applications, there is a key limitation for these materials caused by the polymeric nature of matrices. The highest operation temperatures for common matrices (such as epoxy, bismaleimide, and polyimide matrices) do not exceed 300 °С. Since the 1980s, phthalonitrile resins have been known for their extraordinary thermal behavior, however, this is accompanied by poor processability caused by a narrow processing window [1,2,3]. Thus, all mainstream studies have aimed at decreasing the melting points of phthalonitrile resins and broadening their processing windows. Although there are many papers reporting the synthesis of low-melting phthalonitrile oligomers [4,5,6,7,8,9] and monomers [10,11,12,13,14] suitable for injection molding methods, until recently, only composites manufactured from prepregs by the solution technique have been reported [2,3,15,16,17,18]. The recent study by Kepman’s group resulted in the development of phthalonitrile resins suitable for cost-effective injection methods for CFRP manufacturing [19,20,21]. The reported composites retained mechanical properties at elevated temperatures up to 450 °С [21], and can be already considered as a substitution for aluminum and titanium alloys in such applications as jet engine blades, the skin of hypersonic aircrafts, etc. These results would not be possible without the development of new low-melting phthalonitrile monomers, linked by phosphate bridges [21,22].

Since usually long post-curing, in addition to fast pre-curing, is needed to obtain material with the best properties, it is preferable to perform final processing on a free-standing part. In this case, it is very important to know the elasticity modulus at every moment during curing to set up a maximum process rate and to simultaneously avoid shape distortion by gravity. Multiscale computer simulations combining coarse-grained (CG) techniques with full atomistic modelling can provide a tool for predicting macroscopic properties of the resulting material from the chemical structure and the way of processing of its constituent monomers. Macroscopic mechanical properties were studied with the aid of computer simulations on many various subjects, from metal-organic frameworks for hydrogen storage [23] to spiderwebs [24]. However, a similar study of polymer compounds is still rather complicated, as a highly crosslinked polymer matrix is a giant network with a sophisticated irregular topology, which is tricky to reproduce and analyze in a relevant way [25,26,27]. Moreover, while static mechanical properties have been extensively studied by means of different simulation techniques, the direct full atomistic approach has not been used to predict dynamic mechanical properties on a quantitative level. In our recent papers, we estimated glass transition temperatures (*T_g_*s) of phthalonitrile monomers melts using molecular dynamics (MD) simulations [22,28], and developed a multiscale procedure for generating fully atomistic, highly crosslinked phthalonitrile networks, and applied it to the case of phthalonitrile resin based on the low-melting monomer, bis(3-(3,4-dicyanophenoxy) phenyl) phenyl phosphate (DPPPP) [29,30]. For both monomer blends and networks obtained in low-temperature crosslinking, we found a good agreement of the estimated *T_g_*s with experiments. As the elasticity modulus of a polymer matrix is one of the most important parameters that determine CFRP strength, in this paper, we summarized the development of the multiscale computational methodology connecting the chemical structure of a monomer unit and mechanical properties of a crosslinked matrix. Namely, we estimated the full range of mechanical properties (stress-strain curves, Young’s modulus, E, viscoelastic storage, E′, and loss, E″, moduli) of phthalonitrile networks at various conversion degrees and temperatures, and compared results with corresponding experimental samples.

Here, we again used the example of the monomer, DPPPP, as one of the promising examples of thermostable resin basis [30]. At the same time, we believe that the presented methodology could be used without significant changes as a universal manual to perform computational studies of crosslinked polymer matrices constructed from a wide range of compounds and through various crosslinking mechanisms. We note that relatively low modulus values were reported for cured matrices based on low-melting phthalonitriles [5,6,7,8,31], so it is interesting to check the applicability of our approach to the case of the DPPPP monomer in this paper. In this regard, our work represents the next step in the direction of in silico studies of the physical properties of polymer matrices, and establishes a connection between simulations and experimental studies. An important feature of our work, apart from a novel and poorly studied object in the form of phthalonitrile-based resins, is that it is an attempt to investigate both static (E) and dynamic (E′ and E″) mechanical properties, and also their direct comparison with experimentally measured characteristics.

## 2. Methodology

### 2.1. Coarse-Grained Model

We used dissipative particle dynamics (DPD) simulations to model matrix curing of phthalonitrile resins at the mesoscale level and to obtain equilibrated network structures and to study their mechanical properties. DPD is a mesoscale simulation technique suggested by Hoogerbrugge and Koelman [32,33], and developed by Espanol, Groot, and Warren (considering the classical lattice Flory-Huggins theory) for simulation of polymers and molecular systems [34,35]. In this method, polymer molecules are represented in terms of the bead-and-spring model, with particles governed by Newton’s equations and interacting through a conservative force (repulsion), a dissipative force (friction), and a random force (heat generator). A soft repulsive potential enhances the stability of the integration of the equations of motion, allows an increase of the time step (in comparison with molecular or Brownian dynamics), and, thus, access to large time and spatial scales in the simulation of complex polymer networks. Recently, this method was successfully adopted for simulating chemical reactions by the concept of “mesoscale chemistry” [25,29,36], and was used to describe the mechanical properties of filled elastomer nanocomposites [37]. We omit the details of this method because they are well described in the publications mentioned. In addition, a recent review and comparison of different molecular scale simulations on thermoset polymers can be found in Ref. [38].

In this work, we studied networks based on the monomer, bis(3-(3,4-dicyanophenoxy) phenyl) phenyl phosphate, and the diamine curing agent, 1,3-bis(4-aminophenoxy)benzene (APB), as an initiator, which continued our recent studies of these systems [22,28,29,30]. Our simulation scheme consists of several sequential processes “Mapping of atomistic structures of comonomers onto equivalent CG representations” → “DPD-curing” → “Reverse mapping of CG models to atomistic ones” → “MD relaxation”. We used coarse-grained and atomistic models to extract mechanical properties at the corresponding simulation levels. Comparative analysis of the obtained results makes it possible to gain important experience in using coarse-grained simulations to evaluate trends in the behavior of materials at various parameters.

**Figure 1:**
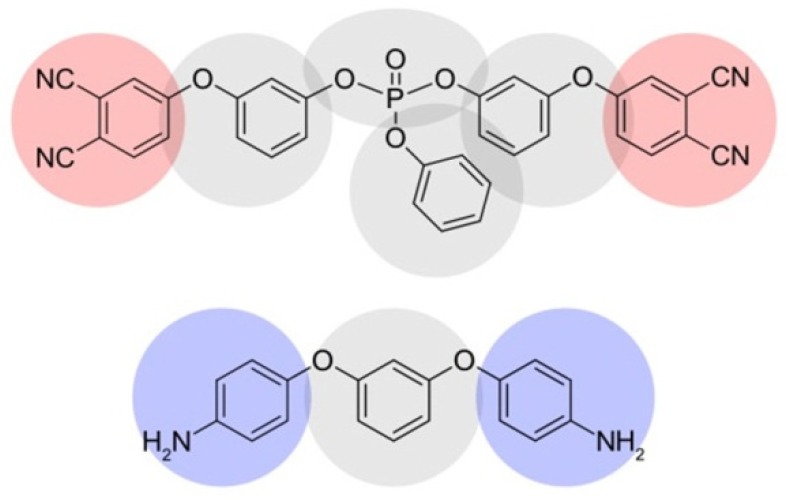
Chemical structures of the phthalonitrile monomer, bis(3-(3,4-dicyanophenoxy) phenyl) phenyl phosphate (DPPPP), and the initiator (diamine curing agent 1,3-bis(4-aminophenoxy)benzene, APB) with their coarse-grained mapping schemes. Red and blue beads have valences of two and one, respectively.

The scheme of the direct mapping of initial chemical structures into the coarse-grained representation is shown in Figure 1. All beads were supposed to have approximately the same excluded volume and the same corresponding DPD interaction parameter, a = 90 (details of the choice can be found in Ref. [30]), at *k*_B_*T* = 1. Additional information about the mapping of atomistic structures of comonomers onto equivalent CG representations and about scaling DPD units to real units is presented in the Supporting Information. The initial simulation boxes of two sizes were chosen. The smaller box of 10 × 10 × 10 DPD units (which, for example, for the system of the conversion degree, 0.1, at *T* = 300 K, corresponded to approximately 7.4 × 7.4 × 7.4 nm^3^) contained 480 monomers, and was used in reverse mapping to obtain the full atomistic model for MD simulations. The larger one of 30 × 30 × 30 DPD units contained 12,960 monomers, and was used solely for DPD simulations. In both cases, the mass proportion of the monomer and initiator molecules was 96:4. Terminal beads of each monomer and initiator (marked red and blue in Figure 1) had free valences equal to two and one, respectively. Terminal beads could be inactive (by default for monomer) or active (by default for initiator). Active sites could form new bonds with inactive terminal beads, and pass the active state to the bead they formed a link with, which mimicked the propagation process in radical polymerization reactions.

### 2.2. Simulation of Curing Process at CG Level

**Figure 2:**
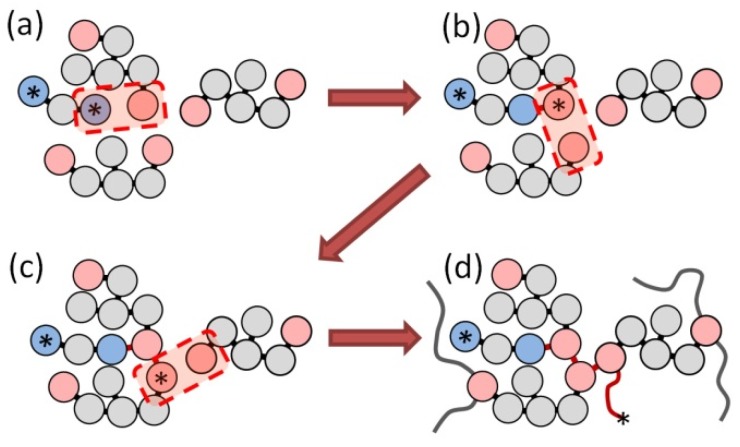
Schematic representation of coarse-grained reactions of initiation (**a**,**b**) and polymerization (**b**–**d**). Terminal beads of each monomer (red) and initiator (blue) have a nonzero valence; asterisk indicates an active bead. Red arrows show the reaction pathways.

The model of the curing procedure was based on the concept of “mesoscale chemistry”, described in previous work [29]. Two types of reactions were implemented at the coarse-grained level:

1. Initiation reaction, when terminal beads of the initiator and monomer molecule form a bond and the corresponding monomer particle takes an active state from the initiator terminal bead (asterisk in Figure 2a,b); and
2. Simple polymerization reaction, when an active site of the monomer forms a new bond with a neighboring terminal bead, and passes the active state to that bead, Figure 2b,c (this process could proceed, as depicted in Figure 2c,d).

These reactions were carried out probabilistically under NVT conditions (constant number of particles, volume and temperature). At regular time intervals, the distance between an active terminal bead and adjacent terminal beads with free valences was compared to the reaction radius, *R_c_* = 1. If it was less than the reaction radius, a new bond between such a pair of beads was formed, with the probability of *p* = 0.01, which is small enough to keep quasi-equilibrium conditions in the vicinity of the reaction centers [36].

It should be noted that this methodology is identical to the one used in our previous works [29,30]. Detailed information on the full atomistic reaction pathways and the corresponding coarse-grained reactions are shown in Figure S3 of the Supporting Information. The studies of the reaction pathways in phthalonitrile-based compounds have shown that the triazine formation is also possible at high temperatures [39,40]. In this paper, we neglect the triazine formation, as it only has a subtle effect on the matrix density, and thus should not change the mechanical properties in the glassy state significantly [30].

During the curing process simulations, the conversion degree was calculated as the ratio between the current amount of bonds between terminal beads and the maximum amount of them (1000 for the smaller box and 27,000 for the larger box).

### 2.3. Mechanical Properties of CG Networks

The mechanical properties of the CG matrices at various conversion degrees were analyzed by DPD simulations of sample deformation. We applied a uniaxial deformation along the X-axis to the simulation box keeping the volume of the box constant and measured stress–strain response curves. Deformations were applied in two steps: (1) Gradual deformation of the system, during which the positions of the beads were changed affinely until the desired strain, λ = $L/L_{0}$, was reached, where *L* and *L*_0_ are the current and equilibrium sample lengths in the direction of elongation, respectively; (2) long relaxation at the fixed strain. The components of the stress tensor, p, were averaged using the virial theorem [37]. The true stress, t, was calculated as t = 〈p_xx_〉 − 0.5〈p_yy_ + p_zz_〉. The sample stiffness, *E*_DPD_, was estimated by the linear fit of equation, t ≈ *E*_DPD_·λ, in the range of small deformations (λ ≤ 1.1).

### 2.4. Reverse Mapping of CG Networks

The resulting 10 × 10 × 10 DPD units CG systems with conversion degrees from 0.1 to 0.9 contained a skeleton model of crosslinked polymer networks, and were used as the input for our reverse mapping procedure. This procedure extracts the following information of the final state of the CG system, viz., types of coarse-grained particles, their coordinates, and all bonds between them (intramonomer and newly formed). The implementation of the reverse mapping procedure is described in Ref. [30]. After the reconstruction of the atomistic structure, the additional procedure was applied to assign the correct types of all atoms in pcff [41] notation. This information makes it possible to calculate all the partial charges (according to the rules defined in the pcff force field), and, together with the topological analysis, can be used to select the proper constants of intramolecular and intermolecular interactions (bond, angle, dihedral and improper potentials, non-bonded 9-6, and Coulomb potentials). See Supporting Information for more details.

### 2.5. MD Simulations of Physical Properties of Atomistic Networks

Then, the glass transition temperatures and mechanical properties of that created in the previous sections’ fully atomistic phthalonitrile networks (of various degrees of conversion, without triazine) were analyzed through molecular dynamics simulations using GROMACS [42,43] and LAMMPS [44] packages and pcff force field for interatomic interactions. A detailed description of the equilibration procedure, simulation parameters, and calculation of *T_g_*s is given in our previous work [30]. Shortly, all molecular dynamics simulations were performed in a constant temperature and constant pressure (NPT) ensemble at normal pressure *p* = 1 atm. The systems were firstly equilibrated for about 23 ns at temperatures that varied in the range of 300–650 K (with timestep of 0.01 fs–1 fs) in LAMMPS; and all of the subsequent equilibration and production MD runs were performed using the GROMACS engine (with a time step of 1 or 2 fs, depending on the system). The systems were equilibrated for 30 ns at *T* = 600 K, for 30 ns at *T* = 400 K, for 30 ns at *T* = 800 K, cooled down to *T* = 100 K at a cooling rate of 10 K/ns with a step of 100 K, heated up to *T* = 800 K at a heating rate of 10 K/ns with a step of 100 K, and equilibrated for 20 ns at *T* = 800 K. For estimation of *T_g_*s, the systems were cooled down from *T* = 800 K to *T* = 100 K at a cooling rate of 5 K/ns and a cooling step of 20 K. *T_g_* was calculated from the change in the slope in the density-temperature dependencies at temperatures of 100–700 K. In this work, for further mechanical analysis, all of the systems were additionally equilibrated separately for 30 ns at *T* = 300 K, *T* = 450 K, and *T* = 600 K, and, then, for each system at each temperature, 10 independent starting conformations were saved every 0.1 ns (during 0.9 ns). For the mechanical analysis, we also considered systems at *T* = 300 K, which were cooled down from *T* = 800 K to *T* = 300 K slower at a cooling rate of 5 K/ns and a cooling step of 20 K. The equilibrated networks contained 32,240 atoms, and at a normal pressure, *p* = 1 atm, and temperature, *T* = 600 K, had dimensions from 7.4 × 7.4 × 7.4 nm^3^ to 7.8 × 7.8 × 7.8 nm^3^, depending on the degree of conversion. During production runs, the trajectory was saved every 5 or 10 ps (depending on the system) for the calculation of *T_g_*s, and every 0.2 ps for the calculation of the mechanical properties.

The mechanical properties of the resulting equilibrated networks were analyzed by applying small amplitude uniaxial deformation (either with a constant deformation rate or cyclic) to the simulation box, and measuring its mechanical response. The pressure was fixed using Berendsen semiisotropic barostat [42], with the compressibility set to 4.5·10^−5^ bar^−1^ and 0 bar^−1^ in the X/Y directions and in the direction of elongation Z, respectively. During the deformation, the stress-strain curves were plotted, with the strain defined as $\varepsilon \left(t\right)=\left(L\left(t\right)-L_{0}\right)/L_{0}$, where L(t) and $L_{0}$ are the instant and initial sizes of the simulation box in the direction of elongation, respectively, and stress, σ(t), was calculated as the negative value of the diagonal component of the pressure tensor, corresponding to the direction of elongation, -p_zz_.

First, the systems were uniaxially elongated by approximately 3% (up to strain of ~0.03, i.e., in the linear viscoelastic regime of deformation) with constant deformation rates of *v* = 5·10^−6^ nm/ps, 1·10^−5^ nm/ps, 5·10^−5^ nm/ps, 1·10^−4^ nm/ps, 5·10^−4^ nm/ps, 1·10^−3^ nm/ps along Z directions for systems at *T* = 300 K and *T* = 600 K, and along X-, Y-, or Z directions for systems at *T* = 450 K, that corresponds to the strain rates, $\dot{\varepsilon }=v/L$, from about 7·10^5^ s^−1^ to about 1·10^8^ s^−1^. For the demonstration of the reversibility of the static deformation, the system of the conversion degree, 0.9, at *T* = 300 K, deformed along Z direction, was also deformed in the opposite direction (that is, with a rate, *v* = –5·10^−5^ nm/ps) to the initial state. For simulation of elongation in the X- and Y- directions, the systems were rotated so that the axis, X- or Y, correspondingly, was placed instead of axis Z. The Young’s modulus, E, was then calculated from the slopes of the linear fits of the initial (up to 1.5% of deformation) parts of the stress-strain curves (in the viscoelastic regime of deformation) using the method of least squares. For each case, the deformation was performed for 10 independent starting conformations of the same system, prepared on the stage of equilibration, and the resulting Young’s modulus (and its standard deviation) was calculated by averaging over these 10 MD runs, and, for the systems at *T* = 450 K, over 3 elongation directions.

**Figure 3:**
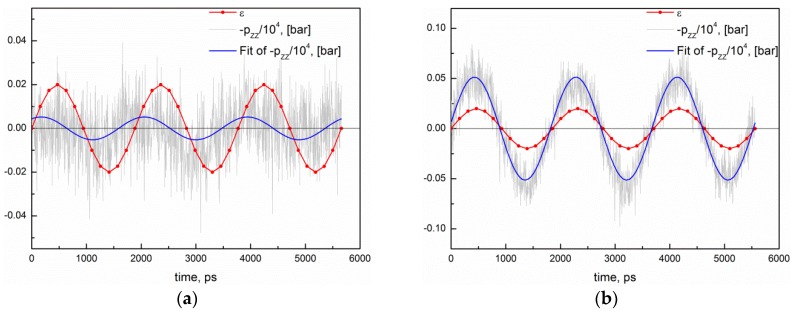
Determination of the storage and loss moduli for systems at *T* = 450 K: (**a**) The case of a viscous medium, when E″ > E′ for the system at the conversion degree, 0.1, (E′ = 0.14 GPa, E″ = 0.22 GPa, tan *δ* = 1.56182), and (**b**) the case of elastic medium, when E′ > E″ for the system at the conversion degree 0.9 (E′ = 2.55 GPa, E″ = 0.31 GPa, tan *δ* = 0.12104). The oscillation frequency varied in the range of 5.3·10^8^–5.4·10^8^ Hz.

Then, the elastic (storage) and viscous (loss) moduli (viscoelastic properties) were analyzed by applying uniaxial cyclic elongation with maximum strain, $\varepsilon_{0}$, equal to 0.02 and maximum deformation rate of 5·10^−4^ nm/ps along the Z direction to the systems of various conversion degrees at *T* = 450 K. This corresponded to the oscillation frequency, which varied in the range of 5.3·10^8^–5.4·10^8^ Hz. To check the influence of the oscillation frequency on the results, we additionally simulated the same cyclic deformations, but with maximum deformation rates equal to 2.5·10^−4^ nm/ps and 1·10^−3^ nm/ps (and oscillation frequencies equal to 2.7·10^8^ and 1·10^9^ Hz, respectively) for the system of the conversion degree, 0.9, at *T* = 450 K. The sinusoidal strain, $\varepsilon \left(t\right)=\varepsilon_{0}\sin\left(wt\right)$, where w is the frequency of oscillations, was mimicked by the stepwise deformations with constant rates. Namely, the complete deformation cycle was divided on 12 equal time intervals, and at each interval the system was deformed at a constant rate with such a value that the strain in the end of the interval was the same as if the deformation was sinusoidal. The stress response was measured during 3 complete cycles of deformation, and fitted by the equation, $\sigma \left(t\right)=\sigma_{0}\sin\left(wt+\delta \right)$, where δ is the phase difference between the stress and strain (Figure 3). The storage modulus, $E^{\prime }=\left(\sigma_{0}/\varepsilon_{0}\right)\cos\left(\delta \right)$, the loss modulus, $E^{\prime \prime }=\left(\sigma_{0}/\varepsilon_{0}\right)\sin\left(\delta \right)$, and the mechanical loss coefficient (or damping coefficient), $\tan\delta =E^{\prime }/E^{\prime \prime }$, were then determined (Figure 3).

## 3. Experimental

Monomer DPPPP was obtained as described in Ref. [22]. Initiator APB was purchased from Sigma Aldrich and used as received.

To obtain molded plates, the monomer DPPPP (50 g) was placed into a 250 mL flask, then melted and degassed by stirring under vacuum at 140 °C until a homogeneous dark resin was obtained. Then, APB (2 g) was added and the mixture was stirred for 20 more minutes at 300 RPM at 140 °C. Next, the mixture was poured into a metal mold for dynamic mechanical analysis (DMA) samples (100 × 100 × 2 mm). The molds were placed into an air circulated heated oven and cured at 200 °C for 6 h. Next, the molds were disassembled and the cured plates were cut by milling on a CNC machine according to the measurement requirement, and samples were post-cured under inert atmosphere at 250 °C, 300 °C, and 350 °C for 3 or 6 h at each temperature, with a heating rate of 2 °C/min between holds.

DMA was performed using TA Instruments DMA Q800 in a three-point bending regime with a varied frequency and amplitude of 40 μm, with a heating rate of 5 K/min. The glass transition of the cured samples was measured by dynamic mechanical analysis and assigned according to ASTM E1640 with accuracy ±4 °C. To measure E′ and E″, a calibration of the apparatus was performed according to ASTM E2254 on four standard carbon steel samples of different thicknesses. E′ and E″ were measured with a precision of 4.0%.

## 4. Results and Discussion

In this section, we present the simulations’ results (mechanical properties) for standard phthalonitrile resin based on monomer DPPPP and initiator APB (see Figure 1), and compare them with the obtained experimental data.

**Figure 4:**
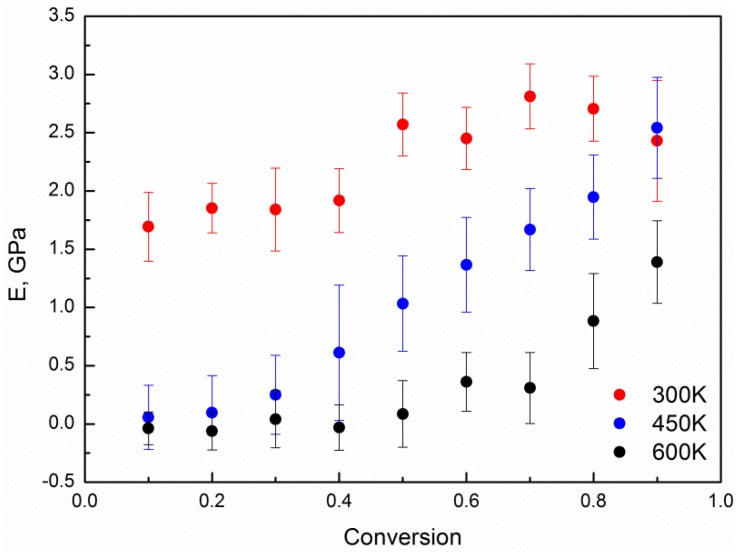
The Young’s modulus of systems at temperatures *T* = 300 K, 450 K, and 600 K, deformed with a rate of 5·10^−5^ nm/ps along the Z direction, as a function of the conversion degree.

At first, we measured the mechanical response of atomistic phthalonitrile networks of various conversion degrees at various temperatures to uniaxial deformation with a constant rate (in the linear viscoelastic regime of deformation) using MD simulations. The stress was calculated as the negative value of the diagonal component of the pressure tensor, corresponding to the direction of elongation, and the Young’s modulus was then determined from the resulting stress-strain curves. The typical example of strain dependencies of the diagonal components of the pressure tensor (p_xx_, p_yy_, -p_zz_) for the system of the conversion degree, 0.9, at *T* = 300 K deformed with a rate of 5·10^−5^ nm/ps along the Z direction, before averaging, is shown in Figure S4a. The strain dependencies of the diagonal component, -p_zz_, for this system deformed with a rate of 5·10^−5^ nm/ps along the Z direction and along the opposite direction to the initial state (that is, with a rate, v = –5·10^−5^ nm/ps), as shown in Figure S4b, demonstrate the reversibility of static deformation. The Young’s modulus increases with the deformation rate (see Figure S5), and stays approximately the same if the direction of elongation was changed (see Figure S6). For our mass calculations, we have chosen the deformation rate optimal in terms of consuming CPU hours—5·10^−5^ nm/ps, as the deformation rates less than 10^−4^ nm/ps lead to close values of the Young’s modulus (see Figure S5). The Young’s modulus, as a function of the conversion degree at various temperatures, is shown in Figure 4. The calculated Young’s modulus at *T* = 300 K only slightly increases with conversion and varies in the range from 1.7 GPa to 2.8 GPa. At higher temperatures, the increase of the modulus with conversion is more pronounced (at *T* = 450 K—from 0.1 GPa to 2.5 GPa, at *T* = 600 K—from ~0 GPa to 1.4 GPa) and starts at higher conversion degrees (for systems at *T* = 450 K—at conversion degree ~0.2, for systems at *T* = 600 K—at conversion degree ~0.4, see Figure 4). This is in a good agreement with the glass transition temperatures of these systems calculated in our previous work [30]. At *T* = 300 K, all of the systems are glassified, at *T* = 450 K, the systems are glassified at conversion degrees higher than 0.5, and all of the systems have *T*_g_s less than *T* = 600 K. The Young’s moduli of the systems at *T* = 300 K, which were cooled down from *T* = 800 K to *T* = 300 K slower, was only slighter larger (on average, on ~0.4 GPa) than for the systems with fast cooling. In general, this behavior, at a qualitative level, is quite understandable and shows that at low and high temperatures the contribution of crosslinking of the matrix into a single network into the modulus is rather limited, and is most pronounced for intermediate temperatures. For such temperatures, the modulus increases both due to the appearance of a continuous network of covalent bonds (starting from the conversion degree of 0.2, which is also confirmed by Figure S7, where the dependence of the size of maximum cluster on the conversion degree is shown), and due to freezing of the degrees of freedom and lowering of the total glass transition temperature during crosslinking of the sample.

**Figure 5:**
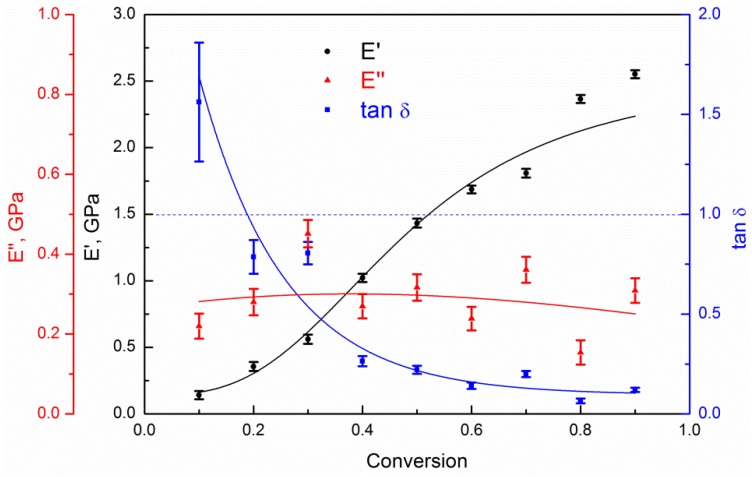
The dynamic mechanical moduli, E′, E″, and mechanical loss coefficient, tan δ, for systems at *T* = 450 K as a function of the conversion degree. The oscillation frequency varied in the range of 5.3·10^8^–5.4·10^8^ Hz.

Next, we considered the dynamic mechanical characteristics, namely E′, E″, and the mechanical loss coefficient (or damping coefficient), tan δ, which is the E′/E″ ratio, at a fixed temperature of 450 K and frequency varied in the range of 5.3·10^8^–5.4·10^8^ Hz, as shown in Figure 5. The values of the storage modulus (similar to the values of the Young’s modulus) smoothly rise from about 0 to 2.5 GPa as the conversion degree increases. The dependence of the loss modulus on the conversion degree is much less pronounced and is determined with a sufficiently large error. In general, we can state a weak extremum, with a maximum in the sol-gel transition region (near the conversion degree equal to 0.2), and with average values of about 0.3 GPa. The corresponding tan δ demonstrates typical behavior for crosslinking systems. The transition from viscous behavior at small conversion degrees (tan δ > 1.0) to elastic behavior (tan δ < 1.0) at large conversion degrees is in full accordance with classical rheological literature data [45]. We note that the point of the sol-gel transition, defined from rheological data as tan δ = 1.0 (the blue dashed line in Figure 5), corresponds to a conversion degree of about 0.2, which coincides well with the sol-gel transition determined from the maximum cluster (see Figure S7, and also Ref. [30]).

**Figure 6:**
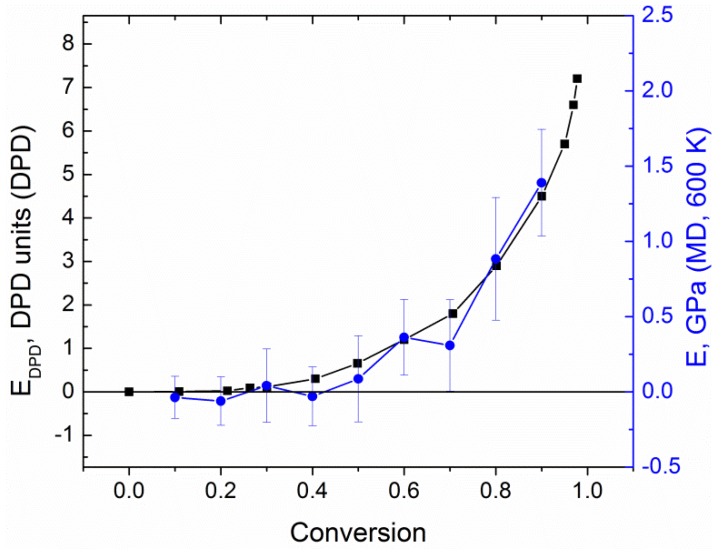
Comparison of the moduli of elasticity in dissipative particle dynamics (DPD) and molecular dynamics (MD) simulations (at *T* = 600 K). The relation between the two scales (DPD and MD) was chosen by fitting these two datasets with a fixed zero level.

The static mechanical properties estimated from MD simulations were then compared with the data obtained by DPD simulations. Glassification of the samples does not appear in our DPD model, thus, we compared the DPD data only with MD results at elevated temperatures of 600 K, which is 100 to 200 K above *T*_g_ depending on the conversion degree. Data on the matrix stiffness at various conversion degrees obtained in DPD is shown in Figure 6 (black squares), with results of MD simulations (blue circles) corresponding to the right scale. One can easily observe that there is a good qualitative matching between both simulation techniques. The system shows zero moduli for the poorly crosslinked system, and elasticity exponentially increases soon after the sol-gel transition (compare with Figure S7). The dimensionless units in DPD were rescaled to GPa by the simple linear scaling procedure, and the resulting scale factor was 0.3 GPa per DPD unit. As a result, the correspondence between DPD units and real units is established for the systems under study and, later, we will be able to measure the mechanic properties of unfrozen samples only by DPD technique.

**Figure 7:**
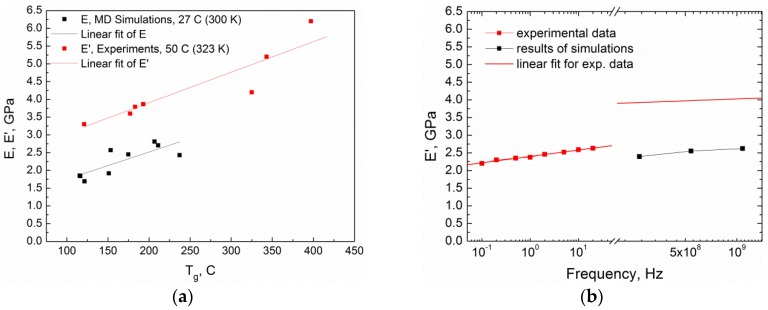
Comparison of the mechanical properties in MD simulations and in experiments: (**a**) The Young’s modulus in MD simulations (at *T* = 300 K) and the storage modulus in experiments (at *T* = 323 K) as a function of the glass transition temperature; (**b**) the dynamic moduli, E′, in MD simulations (for the sample of the conversion degree of 0.9) and in experiments (for the sample post-cured at 300 °C for 6 h) as a function of the oscillating frequency. Both simulations and experimental measurements were performed at 450 K.

Collation of simulated data and experimental results (see Table 1 and Figure 7) is also interesting and very important. However, it is restricted by the obscurity of conversion degrees in the experimental samples. Thus, we compared the matrix stiffness of various samples versus their glass transition temperatures instead of conversion degrees. Figure 7a illustrates the similarity of data from MD simulations and experimental results, where black points correspond to the MD simulations of the Young’s modulus (300 K) and red points correspond to the experimental measurements of the storage modulus (323 K, 1 Hz). Solid lines are estimations of the tendencies in simulations and experiment. As was discussed before in Ref. [30], the range of the observed *T*_g_ in simulations is smaller than the experimental one due to slightly lower densities in simulated systems compared with the available experimental data. For the same reason, the range of the matrix stiffness is also more limited and belongs to lower values of E in our simulations. At the same time, the resulting tendencies (slopes of linear fits of data) in simulations and experiments are coinciding. Thus, we claim a good qualitative agreement between 1 Hz experimental measurements and simulation results for static measurements, while exact values of both the moduli and *T*_g_ are underestimated in computer simulations.

The comparison of the dynamic moduli, E′ and E″, in MD simulations (at a frequency of around 500 MHz) and in experiments (at a frequency of 1 Hz) for the samples at *T* = 450 K is presented in Figure S8. The simulated high-frequency values of both E′ and E″ are surprisingly in good agreement with the obtained experimental low-frequency data throughout the whole range of curing degrees available. To elucidate this coincidence, we performed an additional frequency sweep over an available range both in the experiments (for the sample post-cured at 300 °C for 6 h) and simulations (for one particular sample with the conversion degree of 0.9), as shown in Figure 7b. Unfortunately, there is no possibility of making low-frequency simulations due to limited CPU sources, so we can only compare extrapolated data. Again, black points correspond to MD simulations and red points correspond to the experimental measurements, while the red line extrapolates the experimental low-frequency results to the high-frequency region of the MD simulations (in log scale). We clearly observe twice the decrease in the simulation data compared to experimental extrapolation, while the tendency of the E′ linear growth with the exponential frequency increase is the same. We suppose that the reason of this understatement is the effect already mentioned above of a slightly lower density in our model (see also Ref. [30]). In general, it allows us to use the suggested scheme of simulation to make reasonable predictions of the mechanical properties for certain materials.

## 5. Conclusions

To conclude, here we presented and tested in silico methodology for studies of the static and dynamic mechanical properties of crosslinked polymer matrices on the example of phthalonitrile-derived thermosets. For the first time, the dynamic moduli and mechanical loss coefficient were directly calculated by atomistic molecular dynamics, and compared with experimental data. The results show good quantitative agreement with the experimental DMA measurements at temperatures below and near glass transition. We believe this methodology can be successfully used for predictive simulations for a broad range of polymer matrices in a glassy state, for which the elastic properties almost do not change at a broad range of frequencies.

Also, it was shown that the static mechanical properties at elevated temperatures obtained in atomistic molecular dynamics and dissipative particle dynamics are conformable. This makes it possible to use fast and versatile dissipative particle dynamic simulations for estimations of the mechanical properties of this type of material after the initial mapping of results onto real units. It can greatly improve the accuracy of results for polymer networks with a high structural heterogeneity, as coarse-grained models can reach much larger spatial and temporal scales. We expect that the suggested approach will greatly improve the abilities of computer-aided design of polymers with high-performance thermo-mechanical properties.

## Figures and Tables

**Table 1:** Experimental data on dynamic mechanical analysis (DMA) measurements: Glass transition temperatures and dynamic moduli at 323 K, 450 K, and 600 K for the samples prepared at various curing temperatures and curing times. E′ and E″ were measured at 1 Hz frequency with a relative error bar about 4.0%. *T*_g_ was measured with a precision of 4 °C.

Curing Temperature, °C (K)	Curing Time, hours	E′ @ 1 Hz, MPa			E″ @ 1 Hz, MPa			*T*_g_, °C
		323 K	450 K	600 K	323 K	450 K	600 K	
*Curing*								
200 (473)	6	3300	12.5	–	149	16	–	121
*Post-curing*								
250 (523)	3	3600	984	–	141	262	–	177
250 (523)	6	3790	2170	–	144	206	–	183
300 (573)	3	3865	2150	48.5	190	163	48	193
300 (573)	6	4200	2557	665.6	137	167	141	325
350 (623)	3	5200	4190	2154	205	261	344	343
350 (623)	6	6200	5500	3780	204	261	298	397

## Supplementary Materials

The following are available online at http://www.mdpi.com/2073-4360/10/7/792/s1. A listing of the contents of the file supplied as Supporting Information: description of the atomistic mapping—reverse mapping procedure, scaling DPD units to real units, chemical reaction pathways, comparison of deformation and reverse deformation of the matrix, effect of deformation rate on the Young’s modulus in MD simulations, comparison of the Young’s modulus for various directions of deformation, sol-gel transition during curing process and comparison of the dynamic mechanical properties in MD simulations and in experiments.
