# Supplementary material for: Dynamic and Static Mechanical Properties of Crosslinked Polymer Matrices: Multiscale Simulations and Experiments
Source: Polymers (Basel). 2018 Jul 19;10(7):792. doi: 10.3390/polym10070792 (PMC6403808; doi:10.3390/polym10070792)
Supplement: Supplementary file 1 [file polymers-10-00792-s001.pdf]

Supplementary Materials: Dynamic and Static Mechanical Properties of Crosslinked Polymer Matrices: Multiscale Simulations and Experiments

Daria V. Guseva, Vladimir Yu. Rudyak, Pavel V. Komarov, Boris A. Bulgakov, Alexander V. Babkin and Alexander V. Chertovich

Atomistic mapping – reverse mapping procedure

A scheme showing the mapping of the atomistic structures of the phthalonitrile monomer, bis(3-(3,4-dicyanophenoxy) phenyl) phenyl phosphate, and the initiator (diamine curing agent 1,3-bis(4-aminophenoxy)benzene, APB) onto a coarse-grained (CG) representation is shown in Fig. S1. We choose a benzene ring as the representative structural unit. The selected CG representation makes it possible to keep the degrees of freedom that are responsible for the conformational behavior of comonomers, which is convenient for the subsequent construction of atomistic models using the reverse mapping procedure.

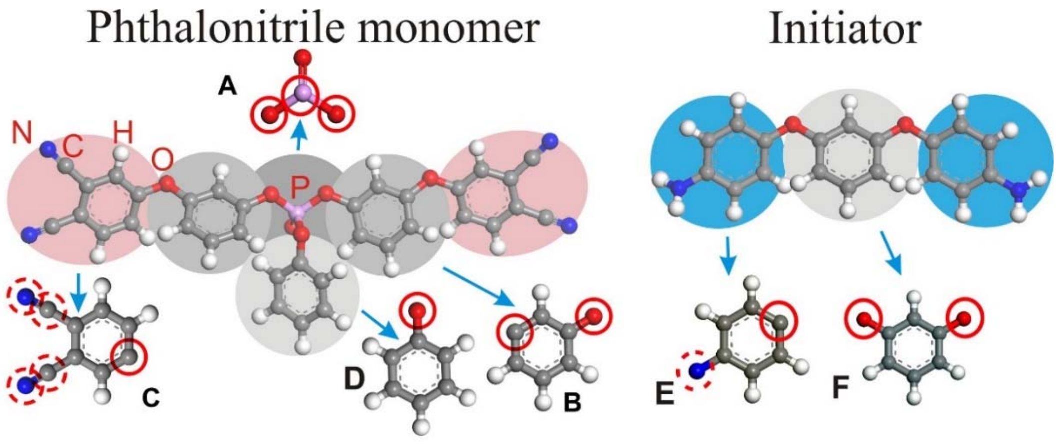

Figure S1. Left picture: chemical structure of the phthalonitrile monomer, bis(3-(3,4-dicyanophenoxy) phenyl) phenyl phosphate, with its CG mapping scheme (the red letters are names of chemical elements). Right picture: the same information for the initiator (diamine curing agent 1,3-bis(4-aminophenoxy)benzene, APB). Six different types of CG particles (beads) are used: A, B, C, D, E, and F.

Six different types of CG particles (beads) are used: A, B, C, D, E, and F. The atomistic structures that correspond to them are used in the reverse mapping procedure. The passive reactive atoms (RA, marked with solid red circles) depict the atoms, which are already connected by covalent bonds in the initial monomers. This information is very important for correct functioning of the reverse mapping procedure. In turn, the active RA (marked with dashed red circles) are used for introducing new chemical bonds that arise between the pairs of beads during the simulation of chemical reactions (see section *Simulation of Curing Process at CG Level*).

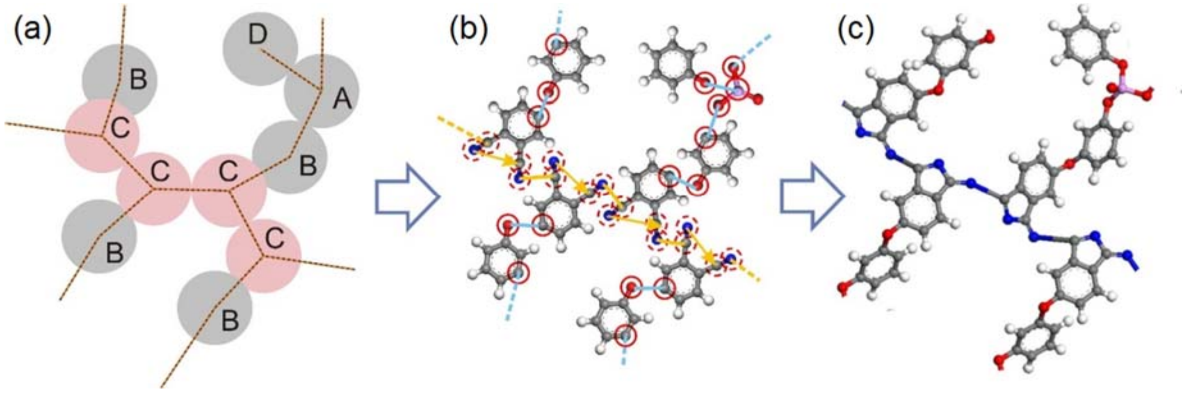

Figure S2. Illustration of the reverse mapping procedure. (a) Fragment of a CG structure (the letters denote different types of CG beads, which are replaced by the corresponding atomistic structures by aligning their centers of mass, Fig. S1). (b) Restoration of intramolecular bonds between passive RA (blue lines) and intramolecular (orange arrows) and intermolecular bonds (orange lines) between active RA. (c) The result of the reverse mapping (the lengths of the new bonds are not relaxed).

To build the full atomistic models of samples of crosslinked matrices, we used the reverse mapping procedure from our previous work [1], which is based on the ideas initially presented in [2, 3]. The reverse mapping procedure uses the information on the final state of the CG system: types of beads, their coordinates, and bonds between them (intramonomer and newly formed), and the atomistic models of chemical structures corresponding to beads (see Fig. S1). It can be represented as a sequence of the following steps:

Step 1. Scaling the coordinates of all beads $\{\mathbf{r}_i\}\rightarrow\{C\mathbf{r}_i\}$ and the size of the simulation cell $L' = CL$ using the scaling factor $C = (M/\varrho)^{1/3}/L$. Here, M is the total mass of the atomistic model, ϱ is the density of the atomistic model, L and L' are the lengths of the edges of the CG and the atomistic simulation cells, respectively.

Step 2. Replacement of beads (see Fig. S2) by the corresponding atomistic structures by aligning their centers of mass, and rotating the substituted atomistic structures to minimize the distances between the passive RAs (the atoms between which chemical bonds, corresponding to the chemical bonds between beads, already existed).

Step 3. Restoring intramonomer chemical bonds between passive RAs and minimization of the bond lengths between them using the Monte-Carlo (MC) procedure from paper [2].

Step 4. Editing the bonds within the beads of the C type (see Fig. S2b) according to the changes in the chemical structure of the monomers during the chemical reaction (see Fig. S3c,d below) and introducing the intermonomer bonds. After that, the bond lengths between all the RAs are re-minimized using the MC procedure.

Step 5. Checking whether all atoms have correct valences. If vacant valences are found in the system, they are filled with hydrogen atoms.

In this way, in steps 1-5 the atomistic structure of the whole system is generated taking into account all the bonds that already existed in the initial monomer structures and that were formed during the crosslinking reaction. As a separate stage, all bonds crossing the rings (problem of spearing) are detected using a procedure for geometric analysis. The detected wrong bonds are eliminated by relocating molecular fragments using Monte-Carlo procedure [1].

In order to correctly build the input files for MD simulation using the valence-force field pcff, it is necessary to make the right choice of the interaction potentials and their parameterization. To do this we define the types of all atoms (in the pcff notation) by analyzing whether an atom belongs to one of the chemical structures defined by the templates in the pcff. The found types of the atoms are used: i) to calculate the partial atomic charges (according to the rules defined in pcff), and ii) along with a topological analysis, to build lists of atoms involved in various types of intramolecular and intermolecular interactions. Using this data, we generate the input files for MD simulations (containing information about the types of atoms and their properties and coordinates, the system topology and the information about the force field (bond, angle, dihedral and improper potentials,

non-bonded 9-6 and Coulomb potentials). It should be noted that we did not take into account in the expression for the total potential energy of the system the cross terms (such as bond-bond, bond-angle, angle-angle, etc.), since they are necessary only in calculations of the vibrational frequencies.

Scaling DPD units to real units

1) The average mass of one monomer in the full atomistic model was equal to $1.014{\cdot}10^{-21}$ g (610.631 g/mol), in the DPD model - 6 DPD mass units (see Fig. S1). Thus, DPD mass unit m was estimated as $1.69{\cdot}10^{-22}$ g (101.772 g/mol).

2) The measured density of the sample in MD simulations was equal to $1.04{\cdot}10^{-21}$ g/nm^3 ($1.04{\cdot}$g/cm^3). Number density in the DPD model was equal to 3 DPD mass units/(DPD length unit)^3 ($3m/\sigma^3$), which is $5.07{\cdot}10^{-22}$ g/(DPD length unit)^3 ($5.07{\cdot}10^{-22}$ g/ σ^3). Thus, DPD length unit σ was estimated as 0.79 nm.

Chemical reaction pathways

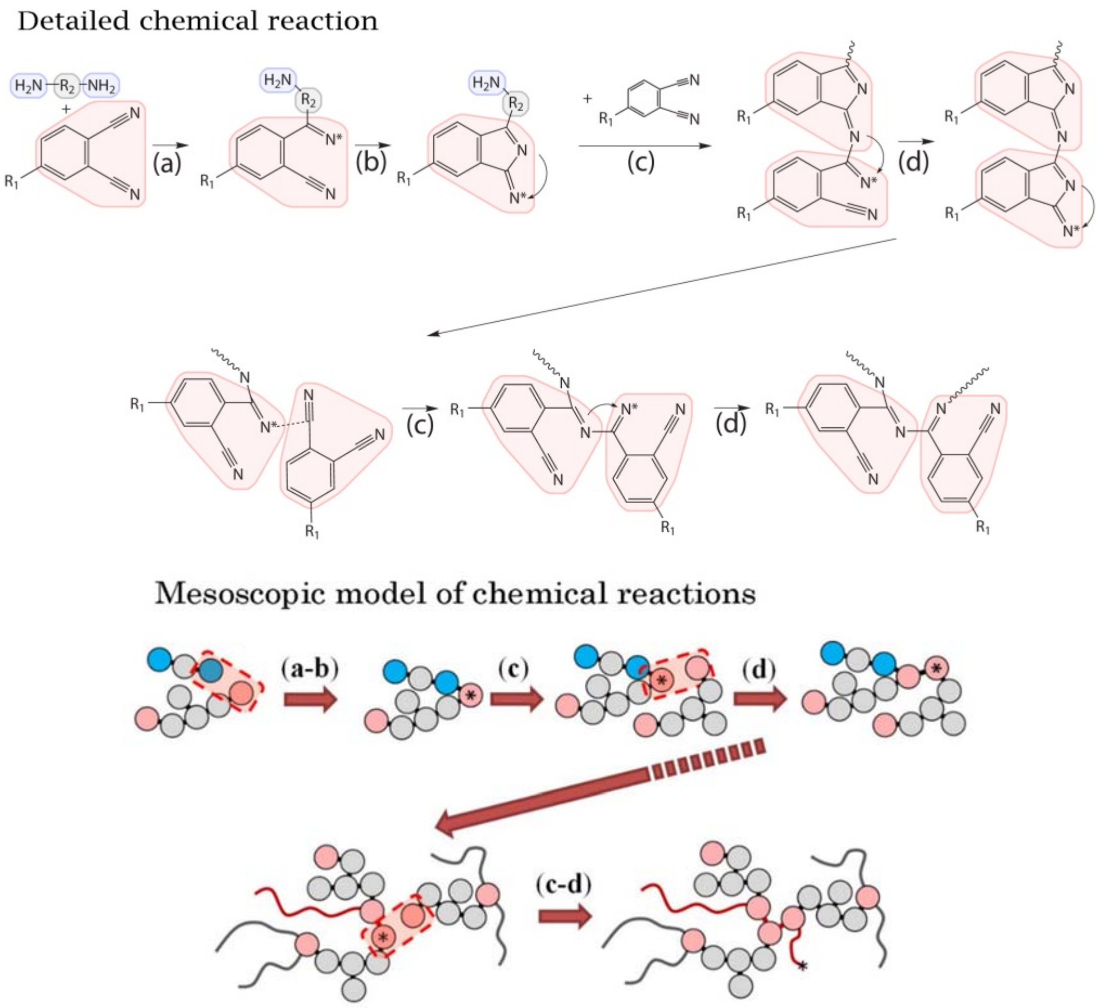

Figure S3. The detailed scheme of chemical reaction (upper picture) and the scheme of chemical reaction in mesoscopic model (lower picture): (a,b) initiation reaction; (c,d) polymerization reaction. Active sites are marked with asterisk. The upper picture shows the transformation of the internal structure of the bead of type C, see Fig. S1.

Comparison of deformation and reverse deformation of the matrix

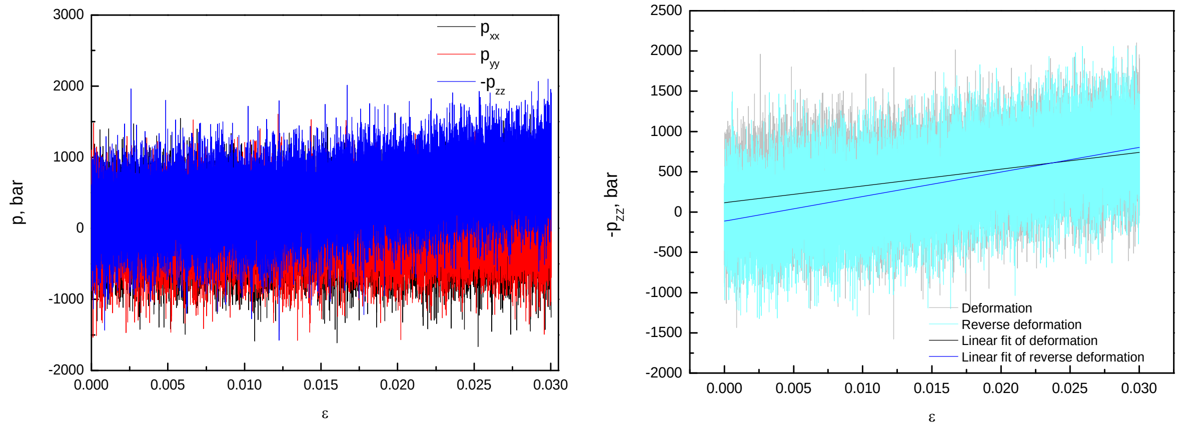

(**a**) (**b**)

Figure S4. (a) A typical example of the realization of uniaxial deformation in the form of the strain dependencies of the diagonal components of the pressure tensor for the system of the conversion degree 0.9 at T = 300 K, deformed with a rate of 5·10^{-5} nm/ps along the Z direction. (b) The strain dependencies of the diagonal component of the pressure tensor, -p_{zz}, for the system of the conversion degree 0.9 at T = 300 K, deformed with a rate of 5·10^{-5} nm/ps along Z direction and along the opposite direction to the initial state (that is, with a rate v = -5·10^{-5} nm/ps) with their linear approximations.

Effect of deformation rate on the Young's modulus in MD simulations

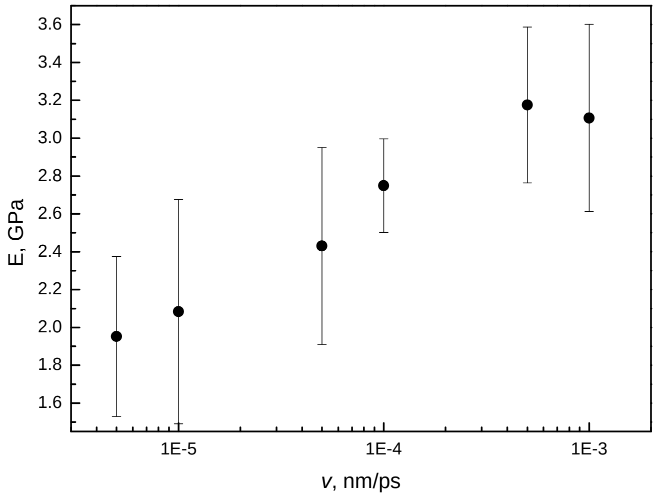

Figure S5. The Young's modulus of the system of the conversion degree 0.9 at T = 300 K as a function of the deformation rate. The deformation was uniaxial linear, up to 3% (see Figure S4), then linear approximations of the initial (up to 1.5% of deformation) parts of the stress-strain curves were made using the method of least squares. The errors were calculated from the results of averaging over 10 MD runs of deformation performed for 10 independent starting conformations of the same system, saved every 0.1 ns.

Comparison of the Young's modulus for various directions of deformation

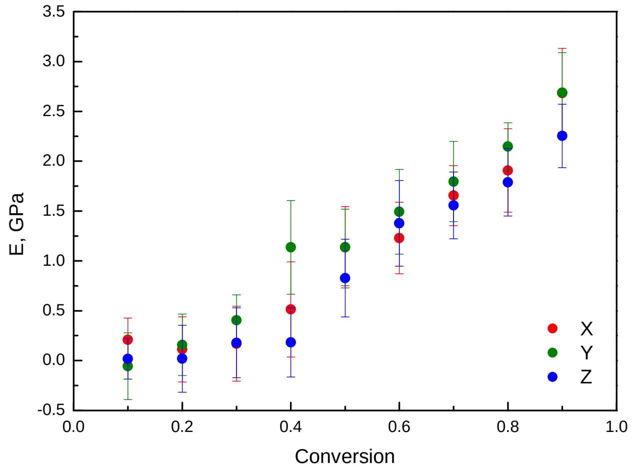

Figure S6. The Young's modulus of the systems at T = 450 K, deformed with a rate of $5{\cdot}10^{-5}$ nm/ps along X-, Y- and Z directions, as a function of the conversion degree.

Sol-gel transition during curing process

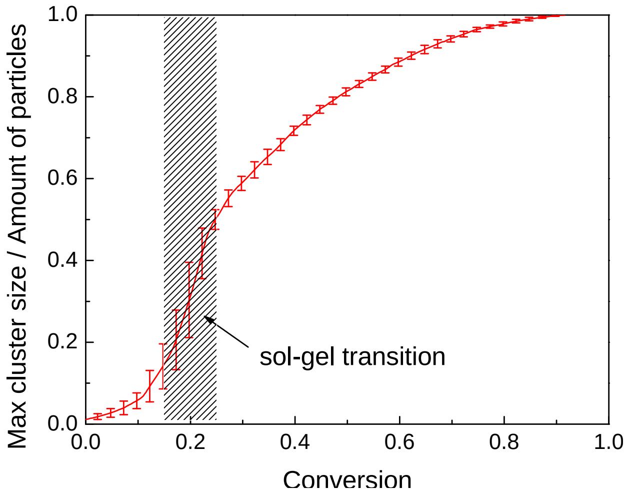

Figure S7. The size of maximum cluster as a function of the conversion degree.

Comparison of the dynamic mechanical properties in MD simulations and in experiments

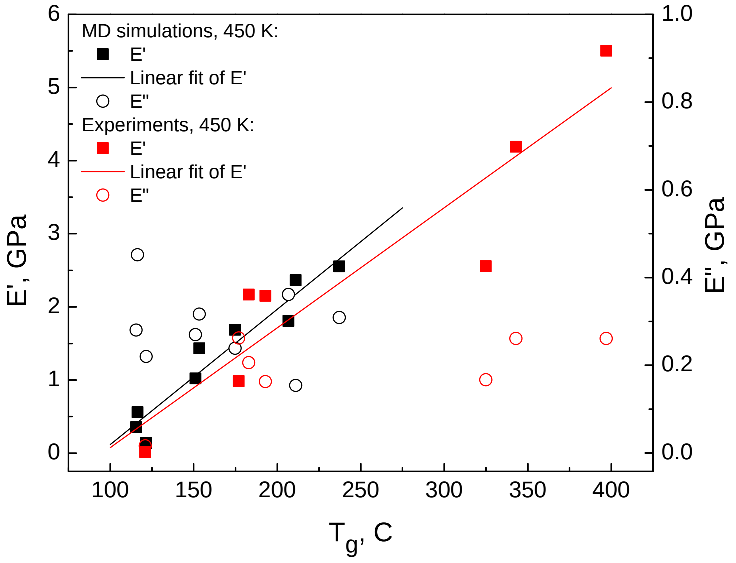

Figure S8. The dynamic moduli, E' and E'', in MD simulations (at frequency varied in the range of $5.3 \cdot 10^8$–$5.4 \cdot 10^8$ Hz) and in experiments (at a frequency of 1 Hz) for the samples at T = 450 K as a function of the glass transition temperature.

References

1. Guseva, D. V.; Rudyak, V. Yu.; Komarov, P. V.; Sulimov, A. V.; Bulgakov, B. A.; Chertovich, A. V. Crosslinkingmechanisms, structure and glass transition in phthalonitrile resins: Insight from computer multiscale simulations and experiments. *J. Polym. Sci. Part B: Polym. Phys.* **2018**, *56*, 362–374, doi: 10.1002/polb.24548.
2. Komarov, P. V.; Yu-Tsung, C.; Shih-Ming, C.; Khalatur, P.G.; Reineker, P. Highly cross-linked epoxy resins: An atomistic molecular dynamics simulation combined with a mapping/reverse mapping procedure. *Macromolecules* **2007**, *40*, 8104–8113, doi: 10.1021/ma070702+.
3. Gavrilov, A. A.; Komarov, P. V.; Khalatur, P. G. Thermal properties and topology of epoxy networks: A multiscale simulation methodology. *Macromolecules* **2015**, *48*, 206–212, doi:10.1021/ma502220k.
